# PGC‐1*α* in hepatic UPR during high‐fat high‐fructose diet and exercise training in mice

**DOI:** 10.14814/phy2.13819

**Published:** 2018-08-14

**Authors:** Caroline M. Kristensen, Maja M. Dethlefsen, Anna S. Tøndering, Signe B. Lassen, Jacob N. Meldgaard, Stine Ringholm, Henriette Pilegaard

**Affiliations:** ^1^ Department of Biology University of Copenhagen Copenhagen Denmark

**Keywords:** Exercise training, high‐fat diet, liver, PGC‐1*α*, UPR

## Abstract

Diet‐induced obesity is associated with hepatic steatosis, which has been linked with activation of the unfolded protein response (UPR). PGC‐1*α* is a transcriptional coactivator involved in exercise training‐induced adaptations in muscle and liver. Therefore, the aim of this study was to test the hypothesis that PGC‐1*α* is required for exercise training‐mediated prevention of diet‐induced steatosis and UPR activation in liver. Male liver‐specific PGC‐1*α* knockout (LKO) and littermate floxed (lox/lox) mice were divided into two groups receiving either control diet (CON) or high‐fat high‐fructose diet (HFF). After 9 weeks, half of the HFF mice were treadmill exercise trained for 4 weeks (HFF+ExT), while the rest were kept sedentary. HFF resulted in increased body and liver weight, adiposity, hepatic steatosis and whole body glucose intolerance as well as decreased hepatic IRE1*α* phosphorylation. Exercise training prevented the HFF‐induced weight gain and partially prevented increased liver weight, adiposity and glucose intolerance, but with no effect on liver triglycerides. In addition, BiP protein and CHOP mRNA content increased with exercise training compared with CON and HFF, respectively. Lack of PGC‐1*α* in the liver only resulted in minor changes in the PERK pathway. In conclusion, this study provides evidence for dissociation between diet‐induced hepatic triglyceride accumulation and hepatic UPR activation. In addition, PGC‐1*α* was not required for maintenance of basal UPR in the liver and due to only minor exercise training effects on UPR further studies are needed to conclude on the potential role of PGC‐1*α* in exercise training‐induced adaptations in hepatic UPR.

## Introduction

Obesity greatly increases the risk of developing metabolic diseases including insulin resistance and nonalcoholic fatty liver disease (NAFLD) (Malnick and Knobler 2006). Excess calorie intake and physical inactivity are the two major contributors to the increasing prevalence of overweight and obesity worldwide (Mayer and Thomas 1967; Booth et al. 2012). Accordingly, studies in rodents have shown that short‐ and long‐term intake of high‐fat diet (HFD) can lead to the development of hepatic steatosis (Koonen et al. 2007; Gollisch et al. 2009; Schultz et al. 2012). In addition, diet‐induced accumulation of triglycerides in liver has in rodents been reported to be associated with glucose intolerance, insulin resistance, hepatic inflammation as well as endoplasmic reticulum (ER) stress with activation of the unfolded protein response (UPR) (Gauthier et al. 2003; Ozcan et al. 2004; Samuel et al. 2004; Gollisch et al. 2009; Chapados and Lavoie 2010; Da Luz et al. 2011; Ren et al. 2012; Deldicque et al. 2013; Wang et al. 2015).

The activation of the UPR in response to HFD is thought to be an attempt to reestablish ER homeostasis (Malhi and Kaufman 2011). Three proximal stress sensors, protein kinase RNA‐like ER kinase (PERK), inositol requiring enzyme‐1*α* (IRE1*α*) and activating transcription factor 6 (ATF6) are held inactive by binding to the chaperone binding immunoglobulin protein (BiP) inside the ER lumen (Bertolotti et al. 2000). In response to ER stress, BiP disassociates from the stress sensors, resulting in dimerization and autophosphorylation of PERK and IRE1*α*, while ATF6 translocates to the Golgi apparatus, where it is cleaved into an active transcription factor. Phosphorylation of IRE1*α* activates its endoribonuclease activity and leads to the splicing of X‐box‐binding protein 1 (XBP1) to the active transcription factor spliced (s)XBP1, whereas activated PERK phosphorylates eIF2*α* resulting in reduced general translation (Harding et al. 1999). Thus, together the three UPR pathways function to dampen ER stress and aim at reestablishing ER homeostasis by decreasing the protein load of the ER, inducing gene transcription of genes involved in protein folding and increasing degradation of misfolded proteins. During conditions where UPR is not sufficient to coping with ER stress and UPR therefore becomes a chronic state, apoptosis rather than cell survival is favored by induction of the proapoptotic CHOP mainly through the PERK pathway (Szegezdi et al. 2006).

Exercise training has been reported to elicit multiple beneficial metabolic effects, including adaptations in the liver. This includes reduction in hepatic triglycerides, inflammation, and insulin resistance in diet‐induced obese rodents (Kawanishi et al. 2012; Alex et al. 2015). Furthermore, the effects of exercise training on diet‐induced ER stress have been examined in rodents (Chapados and Lavoie 2010; Da Luz et al. 2011; Deldicque et al. 2013). Thus, swimming exercise training was shown to prevent a HFD‐induced increase in PERK and eIF2*α* phosphorylation in rat liver (Da Luz et al. 2011), whereas hepatic BiP protein content was reported to increase in HFD fed rats exercise trained on treadmills (Chapados and Lavoie 2010). In addition, exercise training concomitant with HFD increased PERK and sXBP1 protein content in the liver compared with sedentary HFD fed mice (Deldicque et al. 2013). Together this demonstrates contradictory observations on exercise training‐induced regulation of hepatic UPR in rodents on HFD. Moreover, the previous studies examined the effect of exercise training simultaneous with HFD. Hence, the effect of exercise training following a period of HFD on UPR in mice remains to be elucidated.

The molecular mechanisms behind the exercise training‐induced effects on hepatic UPR are still not fully understood. The transcriptional coactivator peroxisome proliferator‐activated receptor gamma coactivator‐1 alpha (PGC‐1*α*) is known to regulate energy metabolism in several tissues, including the liver (Herzig et al. 2001; Yoon et al. 2001; Puigserver et al. 2003). At the basal level, the expression of PGC‐1*α* in liver is low, but a single bout of exercise has been reported to increase hepatic PGC‐1*α* mRNA in mice (Hoene et al. 2009). In addition, PGC‐1*α* was shown to be required for exercise training‐induced metabolic adaptations in mouse liver (Haase et al. 2011). Furthermore, it has been suggested that PGC‐1*α* is involved in mediating exercise‐induced UPR in skeletal muscle through coactivation of ATF6 (Wu et al. 2011). However, whether hepatic PGC‐1*α* is involved in the exercise training‐mediated effects on hepatic UPR during HFD is unknown.

Therefore, the aim of the present study was to test the hypothesis that high‐fat high‐fructose feeding induces hepatic steatosis and activates all three branches of the hepatic UPR and that concomitant exercise training reverses the hepatic triglyceride accumulation and ER stress in a PGC‐1*α*‐dependent manner. A diet with fructose as a source of carbohydrate was used in order to impose a metabolic challenge especially on the liver, because fructose has been shown to be effective in inducing fatty liver (Softic et al. 2016).

## Materials and Methods

### Mice

The liver‐specific PGC‐1*α* knockout (LKO) strain used in this study was obtained by crossing mice homozygous for PGC‐1*α* alleles with exon 3 and 5 flanked by loxP sites (Geng et al. 2010) with mice homozygous for floxed PGC‐1*α* and heterozygous for albumin‐cre (Postic et al. 1999). Littermate floxed (lox/lox) mice were used as controls. The mice were initially genotyped for the floxed PGC‐1*α* alleles and the presence or the absence of the albumin‐cre using PCR on gDNA isolated from an ear piece. Liver‐specific PGC‐1*α* knockout was later confirmed by determining the PGC‐1*α* mRNA level in the liver of all mice (Table 1). Primers used for this are located within exon 3–5 which is deleted in LKO mice (Table 2). All mice received standard rodent chow (Altromin no. 1324; Brogården, Lynge, Denmark) until the beginning of the intervention period and were kept on a 12:12‐h light/dark cycle with lights on 6 am–6 pm. Experiments were approved by the Animal Experiments Inspectorate in Denmark and complied with the European Convention for the Protection of Vertebrate Animals used for Experimental and Other Scientific Purposes (Council of Europe, no. 123 Strasbourg, France, 1985).

**Table 1:** Hepatic PGC‐1*α* mRNA from liver‐specific PGC‐1*α* knockout (LKO) and littermate control (lox/lox) mice fed a control diet (CON) or a high‐fat high‐fructose diet (HFF) for 13 weeks. Half of the HFF mice performed treadmill exercise training the last 4 weeks (HFF+ExT). Values are presented as means ± SE, *n* = 9–10. Primers used are located within exon 3–5 which is deleted in LKO mice

	CON		HFF		HFF+ExT	
	lox/lox	LKO	lox/lox	LKO	lox/lox	LKO
PGC‐1*α* mRNA (exon 4–5)	1.01 ± 0.11	0.01 ± 0.00	0.66 ± 0.10	0.01 ± 0.00	0.87 ± 0.08	0.01 ± 0.00

**Table 2:** Primer and TaqMan probe sequences used for real‐time PCR

Gene	Forward primer	Reverse primer	TaqMan probe
CHOP	5’ CCACCACACCTGAAAGCAGAA 3’	5’ AGGTGAAAGGCAGGGACTCA 3’	5’ CTGGTCCACGTGCAGTCATGGCA 3’
HSP72	5’ GATCACGGTGCCAGCCTATT 3’	5’ CGTGGGCTCATTGATTATTCTCA 3’	5’ TCAGCGGCAAGCCACCAAGGAT 3’
PGC‐1*α* (exon 3–5)	5’ AACCACACCCACAGGATCAGA 3’	5’ TCTTCGCTTTATTGCTCCATGA3’	5’ CAAACCCTGCCATTGTTAAGACCGAGAA3’
sXBP1	5’ TCTGCTGAGTCCGCAGCAGGT 3’	5’ TGCCCAAAAGGATATCAGACTCA 3’	5’ CCCATGGACTCTGACACTGTTGCCTCTT 3’
TNF*α*	5’ ATGGCCCAGACCCTCACA 3’	5’ TTGCTACGACGTGGGCTACA 3’	5’ TCAGATCATCTTCTCAAAATTCGAGTGACAAGC 3’

CHOP, C/EBP homologous protein; HSP72, heat shock protein 72; PGC‐1*α*, peroxisome proliferator‐activated receptor gamma coactivator‐1 alpha; sXBP1, spliced X‐box‐binding protein 1; TNF*α*, tumor necrosis factor *α*.

Another manuscript containing analyses on tissue samples from these mice has recently been published (Dethlefsen et al. 2018).

### Experimental setup

Male LKO and littermate lox/lox mice were at the age of 8–9 weeks divided into two groups, receiving either a high‐fat high‐fructose diet (HFF) or a matched control diet (CON) (D09100304: 20, 40 and 40% of calories from protein, carbohydrates and fat, respectively, and D09100304: 20, 70 and 10% of the calories from protein, carbohydrates and fat, respectively, Research Diets, Inc., New Brunswick, NJ). In the HFF diet, 50% of calories derived from fructose. After 9 weeks of intervention, half of the HFF mice were subjected to exercise training comprising 1 h treadmill running (TSE Systems GmbH, Bad Homburg, Germany) at 15 m/min and 10˚ incline 6 days per week for 4 weeks (HFF+ExT). In addition, the exercise trained mice were adapted to the treadmill by running 10 min twice per day for 5 days prior to the start of the exercise training period. The other half of the HFF mice and the mice on the control diet remained untrained for the following 5 weeks. Additional groups of mice completed the CON, HFF, and HFF+ExT intervention and performed an acute exercise bout at the end of the intervention, but the samples from these mice are not included in this study. Body weight and food intake were registered weekly throughout the intervention period. In addition, all mice were MR scanned (Echo MRI, Echo Medical Systems) 2 days prior to euthanization and percentage body fat was calculated from body weight and amount of body fat (g) obtained from the MR scan. Exercise trained mice performed the last exercise bout 24 h prior to euthanization to prevent any acute effects of exercise. All mice were euthanized by cervical dislocation at ~12 pm–2 pm, trunk blood was quickly collected and liver removed and snap‐frozen in liquid nitrogen. Plasma was obtained by centrifugation of the blood at 2600***g*** and 4°C for 15 min. Liver samples and plasma were stored at −80°C until further analyses. To ensure homogeneity of the liver tissue, liver samples were crushed in liquid nitrogen before analyses.

### Pyruvate and glucose tolerance test

Thirteen weeks into the intervention period and 2 days apart, mice were subjected to first a pyruvate and then a glucose tolerance test. Mice were fasted 18 h and 6 h prior to the PTT and GTT, respectively. A dose of 2 g/kg body weight pyruvate or glucose for the PTT and GTT, respectively, was injected intraperitoneally and blood glucose was measured from tail blood at 0, 15, 30, 45, 60, 90, and 120 min after injection, using a glucometer (Contour, Bayer). PTT and GTT were always initiated at 12 pm. From the resulting data of PTT and GTT, the blood glucose area under the curve (AUC) was calculated using the trapezoid method.

### Plasma metabolites

Plasma free fatty acid concentration was measured using an NEFA‐HR kit according to the manufacturer (WAKO Diagnostics GmbH, Germany).

Plasma triglyceride content was determined as the amount of free glycerol produced by hydrolyzation of triglycerides by ethanolic KOH. From each sample, free glycerol was measured using a Free Glycerol Reagent (Sigma Aldrich, Denmark) leading to the production of a quinoneimine dye that was spectrophotometrically detected at 540 nm in a Multiskan (Multiskan FC, Thermo Scientific). A standard curve was generated from a serial dilution of a Glycerol Standard Solution (Sigma Aldrich, Denmark) and used to convert the absorbance of the samples to a free glycerol concentration. The free glycerol concentration was finally converted to triglyceride content according to the manufacturer.

### Hepatic triglycerides

Triglyceride content was determined on crushed liver samples (~20 mg) using the same procedure as for plasma triglyceride content.

### Hepatic glycogen

Crushed liver tissue (~10 mg) was boiled for 2 h in HCl to hydrolyze glycogen to glycosyl units. After neutralization with NaOH, hepatic glycogen was determined as glycosyl units measured fluorometrically as previously described (Lowry and Passonneau 1972).

### RNA isolation and reverse transcription

Crushed liver (~20 mg) was homogenized for 2 min at 30 s^−1^ using a tissue lyser (TissueLyser II; QIAGEN, Germany) and total RNA was isolated by a modified guanidium thiocyanate‐phenol‐chloroform extraction method (Chomczynski and Sacchi 1987) as previously described (Pilegaard et al. 2000). RNA concentration and purity of samples were determined spectrophotometrically (NanoDrop 1000; Thermo Fisher Scientific). Reverse transcription was performed on 3 *μ*g RNA using Superscript II RNAase H^−^ and oligo‐dT (Invitrogen, Carlsbad, CA) as previously described (Pilegaard et al. 2000).

### Real‐time PCR

Real‐time PCR was performed using ABI‐7900 Sequence Detection System (Applied Biosystems, Forster City, CA) to determine mRNA content of selected genes. Primers and 5’‐6‐carboxyfluorescein (FAM)/3’‐6‐carboxy – N,N,N’,N’‐tetramethylrhodamine (TAMRA)‐labeled Taqman probes (Table 2) were designed using Primer Express 3.0 software (Applied Biosystems) and obtained from TAG Copenhagen (Copenhagen, Denmark). Real‐time PCR was run in triplicates with a reaction volume of 10 *μ*L using Universal Mastermix (Applied Biosystems). The cycle threshold for each sample was converted into an arbitrary amount of target mRNA, using a standard curve generated from a serial dilution of a pooled sample. Total single‐stranded (ss) DNA in each sample was determined, using OliGreen reagent (Molecular Probes, Leiden, The Netherlands) and used to normalize target gene mRNA content as previously described (Lundby et al. 2005).

### Liver lysate

Crushed liver tissue (~25 mg) was homogenized in ice‐cold buffer (10% glycerol, 20 mmol/L Na‐pyrophosphate, 150 mmol/L NaCl, 50 mmol/L HEPES, 1% NP‐40, 20 mmol/L *β*‐glycerophosphate, 10 mmol/L NaF, 1 mmol/L EDTA, 1 mmol/L EGTA, 20 *μ*g/mL aprotinin, 10 *μ*g/mL leupeptin, 2 mmol/L Na_3_VO_4_ and 3 mmol/L benzamidine, pH 7.4) as previously described (Birk and Wojtaszewski 2006) using a tissue lyser (TissueLyser II, QIAGEN, Germany). After 1 h end over end rotation at 4°C, lysates were generated by 20 min centrifugation at 16,000 g and 4°C. Protein concentration was determined by the bicinchoninic acid assay (Thermo Fisher Scientific) and samples were prepared with sample buffer containing sodium dodecyl sulfate (SDS) to yield a protein concentration of 2 *μ*g/*μ*L. Samples were boiled for 3 min at 96°C before protein analyses except for OXPHOS.

### SDS‐PAGE and western blotting

Proteins were separated by SDS‐PAGE and transferred to polyvinylidene fluoride membranes (Immobilon‐P Transfer Membranes; Millipore) by semi‐dry blotting. After blocking in 3% fish gel for 1 h, membranes were incubated overnight at 4°C with primary antibodies against BiP, PERK, PERK^Thr980^ phosphorylation, eIF2*α*, eIF2*α* ^Ser51^ phosphorylation, FAS, JNK, JNK^Thr183/Tyr185^ phosphorylation (#3177, #3192, #3179, #9722, #9721, #3180, #9252 and #9251, respectively, Cell Signaling Technologies, Danvers, MA), IRE1*α*, IRE1*α* ^Ser724^ phosphorylation, OXPHOS, (ab37073, ab48187 and ab110413, respectively, Abcam, Cambridge, UK), PEPCK (10004943; Cayman Chemicals, MI) and ATF6 (#IMG‐273, IMGENEX, San Diego, CA). After incubation with species‐specific horse radish peroxidase conjugated secondary antibody (DAKO, Denmark), the protein or phosphorylation of interest was visualized using an ImageQuant LAS 4000 imaging system and quantified with ImageQuant TL 8.1 software (GE Healthcare, Freiburg, Germany). Protein content and phosphorylation are expressed relative to a pooled control sample loaded in each side of the gel in order to eliminate differences between gels, and to evaluate potential differences across each gel. No effect of genotype or intervention was observed for GAPDH, supporting equal protein content in all samples.

One sample from each experimental group (CON, HFF and HFF+ExT of both genotypes) was loaded on each gel. Because the samples from mice run acutely were run together with the samples used in the present manuscript, the relevant bands have been cut out of the gel pictures for the representative blots. However, the representative blots always show samples run on the same gel (Fig. 6).

### Statistics

All data are presented as mean ± standard error (SE). The presence of main effects of genotype and groups as well as interaction between genotype and groups were tested, using a two‐way analysis of variance (ANOVA). To locate significant differences, the Student Newman Keuls post hoc test was used. If equal variance test failed, data were logarithmically transformed before applying the ANOVA. Throughout, *P* < 0.05 is considered significant and a tendency is reported when 0.05 ≤ *P* < 0.1. Statistical analyses were performed, using SigmaPlot 13.0 (Systat Software, Chicago, IL).

## Results

### Body weight and fat percentage

**Figure 1:**
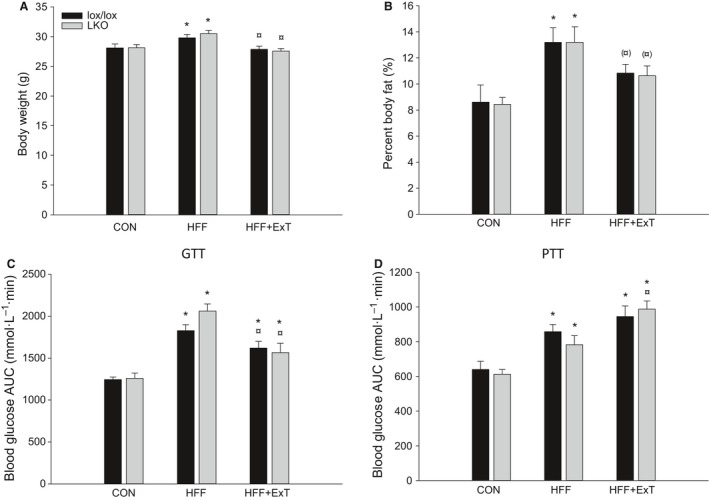
Body weight (A), percent body fat (B), blood glucose area under the curve (AUC) from GTT (C) and PTT (D) from liver‐specific PGC‐1*α* knockout (LKO) and littermate control (lox/lox) mice fed either a control diet (CON) or a high‐fat high‐fructose diet (HFF) for 13 weeks. Half of the HFF fed mice performed treadmill exercise training the last 4 weeks (HFF+ExT). GTT and PTT were performed 1 week before euthanization. AUC is calculated using the trapezoid method. Values are presented as means ± SE,* n* = 8–10. *Significantly different from CON within given genotype, *P* < 0.05. ^¤^Significantly different from HFF within given genotype, *P* < 0.05. Parenthesis indicates a tendency for a difference, 0.05 ≤ *P* < 0.1.

Total body weight was in both genotypes 1.1 fold higher (*P* < 0.05) in HFF than CON mice and exercise training prevented this increase. There was no difference in body weight between genotypes in any of the groups (Fig. 1A). There was no difference in body weight between groups in either of the genotypes or between genotypes at the intervention start (data not shown).

Percentage of body fat was in both genotypes 1.5 fold higher (*P* < 0.05) in HFF than CON mice and percentage body fat tended to be 20% lower in HFF+ExT than HFF (*P* = 0.094 and *P* = 0.054 for lox/lox and LKO, respectively), while there was no difference between CON and HFF+ExT. There was no difference in fat percentage between genotypes in any of the groups (Fig. 1B).

### Glucose and pyruvate tolerance

Area under the curve (AUC) for blood glucose after glucose injection was in both genotypes 1.5 fold and 1.2 fold higher (*P* < 0.05) in HFF and HFF+ExT, respectively, than in CON mice, and AUC was 10–20% lower (*P* < 0.05) in HFF+ExT than HFF. There was no difference in AUC between genotypes in any of the groups (Fig. 1C).

After a pyruvate injection, AUC for blood glucose was in both genotypes 1.3 and 1.5 fold higher (*P* < 0.05) in HFF and HFF+ExT, respectively, than CON mice. AUC was 1.2 fold higher (*P* < 0.05) in HFF+ExT than HFF in LKO mice, whereas there was no difference between HFF and HFF+ExT in lox/lox mice. There was no difference in AUC between genotypes in any of the groups (Fig. 1D).

### Plasma metabolites

There was a tendency (*P* = 0.089) for main a difference between groups in plasma‐free fatty acid concentration, and there was no difference between genotypes (Table 3).

**Table 3:** Plasma free fatty acids and triglyceride concentrations from liver‐specific PGC‐1*α* knockout (LKO) and littermate control (lox/lox) mice fed a control diet (CON) or a high‐fat high‐fructose diet (HFF) for 13 weeks. Half of the HFF mice performed treadmill exercise training the last 4 weeks (HFF+ExT)

	CON		HFF		HFF+ExT	
	lox/lox	LKO	lox/lox	LKO	lox/lox	LKO
Plasma FFA (mg/dL)	0.4 ± 0.1	0.5 ± 0.1	0.4 ± 0.04	0.4 ± 0.05	0.4 ± 0.1	0.3 ± 0.04
Plasma TG (mg/dL)	23.8 ± 7.2	12.7 ± 3.0	46.5 ± 10.6	35.3 ± 5.0^a^	35.0 ± 7.4	28.1 ± 5.9^a^

Values are presented as means ± SE, *n* = 9–10.

a Significantly different from CON within given genotype, *P* < 0.05.

Plasma triglycerides did not differ between groups within lox/lox mice. In LKO mice, plasma triglyceride content was 2.2–2.7 fold higher (*P* < 0.05) in HFF and HFF+ExT than in CON. There was no difference between genotypes in any of the groups (Table 3).

### Liver weight and energy storage

**Figure 2:**
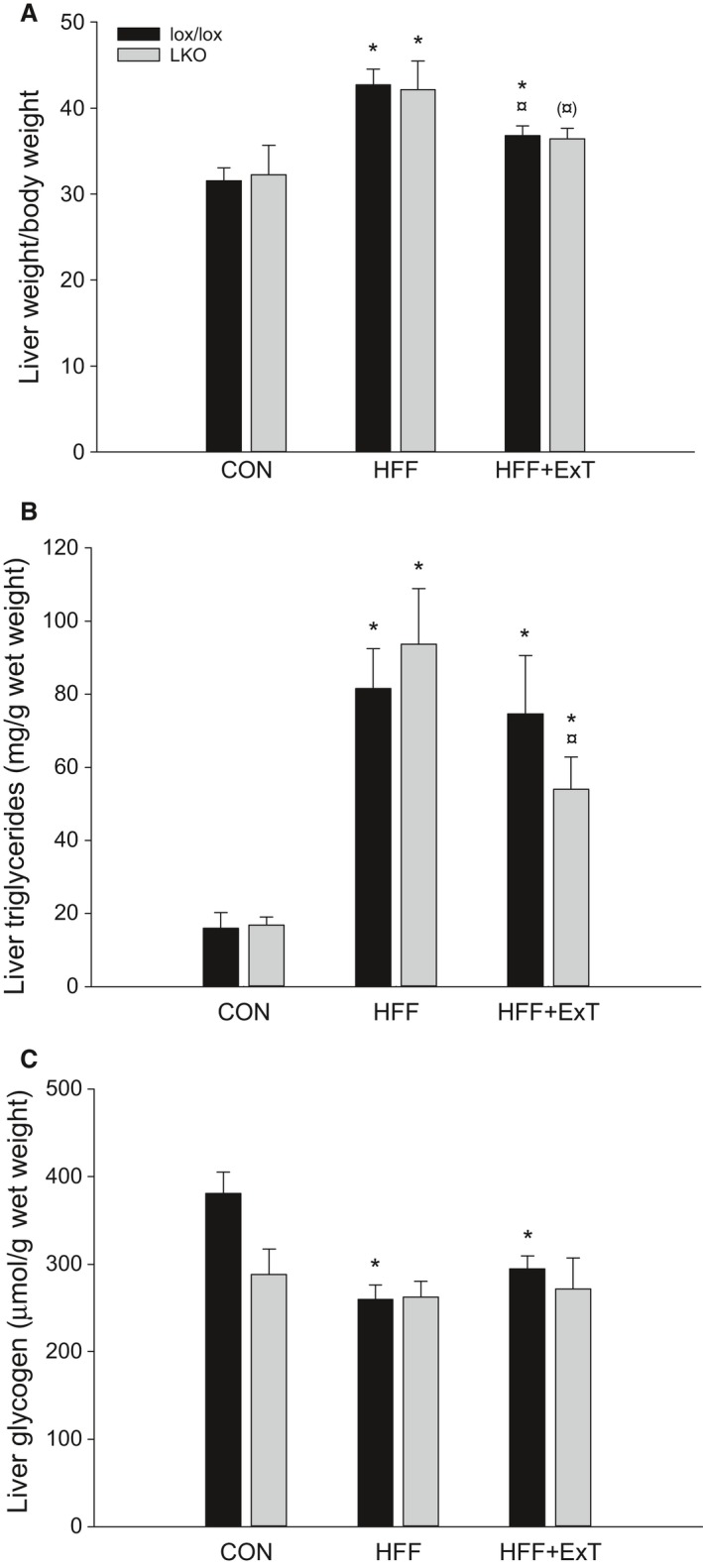
Liver weight (A), triglyceride (B) and glycogen content (C) from liver‐specific PGC‐1*α* knockout (LKO) and littermate control (lox/lox) mice fed either a control diet (CON) or a high‐fat high‐fructose diet (HFF) for 13 weeks. Half of the HFF mice performed treadmill exercise training the last 4 weeks (HFF+ExT). Liver weight is normalized to total body weight. Values are presented as means ± SE,* n* = 9–10. *Significantly different from CON within given genotype, *P* < 0.05. ^¤^Significantly different from HFF within given genotype, *P* < 0.05. Parenthesis indicates a tendency for a difference, 0.05 ≤ *P* < 0.1.

In both genotypes, liver weight normalized to total body weight was 1.4 fold higher (*P* < 0.05) in HFF than CON and in lox/lox mice liver weight was 1.1 fold higher (*P* < 0.05) in HFF+ExT than CON and 15% lower (*P* < 0.05) than HFF, while the liver weight tended to be 15% lower (*P* = 0.093) in HFF+ExT than HFF within LKO (Fig. 2A).

Triglyceride content in the liver was five to sixfold higher (*P* < 0.05) in HFF than CON mice in both genotypes and fivefold and threefold higher (*P* < 0.05) in HFF+ExT than CON in lox/lox and LKO mice, respectively. Moreover, the content of hepatic triglycerides was 40% lower (*P* < 0.05) in HFF+ExT than HFF in LKO mice. There was no difference in liver triglycerides between genotypes in any of the groups (Fig. 2B).

Hepatic glycogen content was 30–35% lower (*P* < 0.05) in HFF and HFF+ExT than CON within lox/lox, while there was no difference between groups within LKO. There was no difference in hepatic glycogen content between genotypes in any of the groups (Fig. 2C).

### PEPCK, FAS and OXPHOS protein

**Figure 3:**
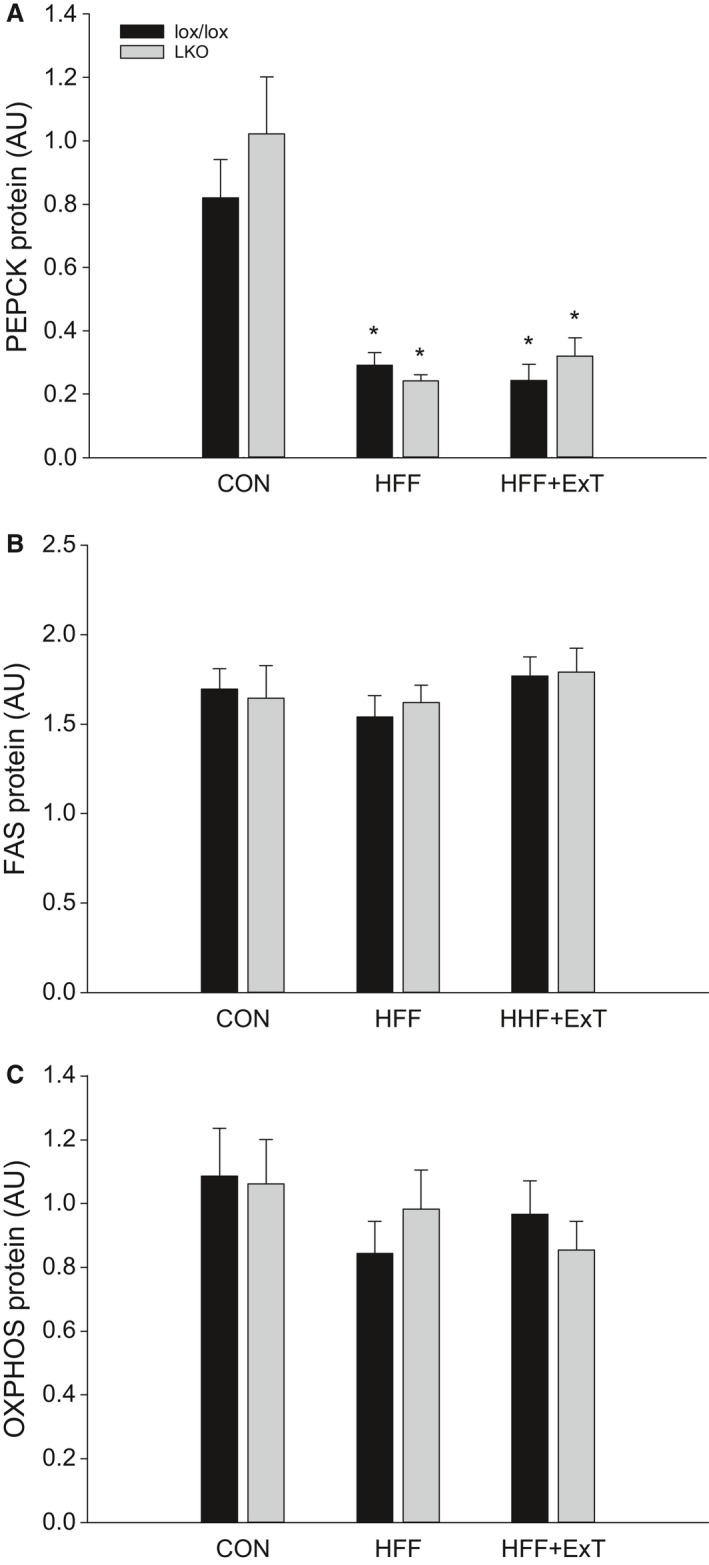
Hepatic PEPCK (A), FAS (B) and OXPHOS (C) protein content from liver‐specific PGC‐1*α* knockout (LKO) and littermate control (lox/lox) mice fed either a control diet (CON) or a high‐fat high‐fructose diet (HFF) for 13 weeks. Half of the HFF mice performed treadmill exercise training the last 4 weeks (HFF+ExT). Protein content is given in arbitrary units (AU). Values are presented as means ± SE,* n* = 8–10. *Significantly different from CON within given genotype, *P* < 0.05.

PEPCK protein content in the liver was 60–80% lower (*P* < 0.05) in HFF and HFF+ExT than CON in both genotypes. There was no difference in hepatic PEPCK protein content between genotypes in any of the groups (Figs. 3A and 6).

Hepatic FAS protein did not change with either HFF or exercise training in any of the genotypes and there was no difference between genotypes in any of the groups (Figs. 3B and 6).

There was no difference in OXPHOS protein between any of the groups and there was no difference between genotypes (Figs. 3C and 6).

### PGC‐1*α* mRNA

Previously reported results from the same study as the present have reported that hepatic PGC‐1*α* mRNA was lower (*P* < 0.05) in HFF than in CON mice and higher (*P* < 0.05) in HFF+ExT than in HFF mice when measured in lox/lox mice (Dethlefsen et al. 2018). In addition, hepatic PGC‐1*α* mRNA was higher (*P* < 0.05) after acute exercise than at rest in HFF mice, while there was no difference between acutely exercised and sedentary lox/lox mice in the HFF ExT group (Dethlefsen et al. 2018).

### UPR proteins

**Figure 4:**
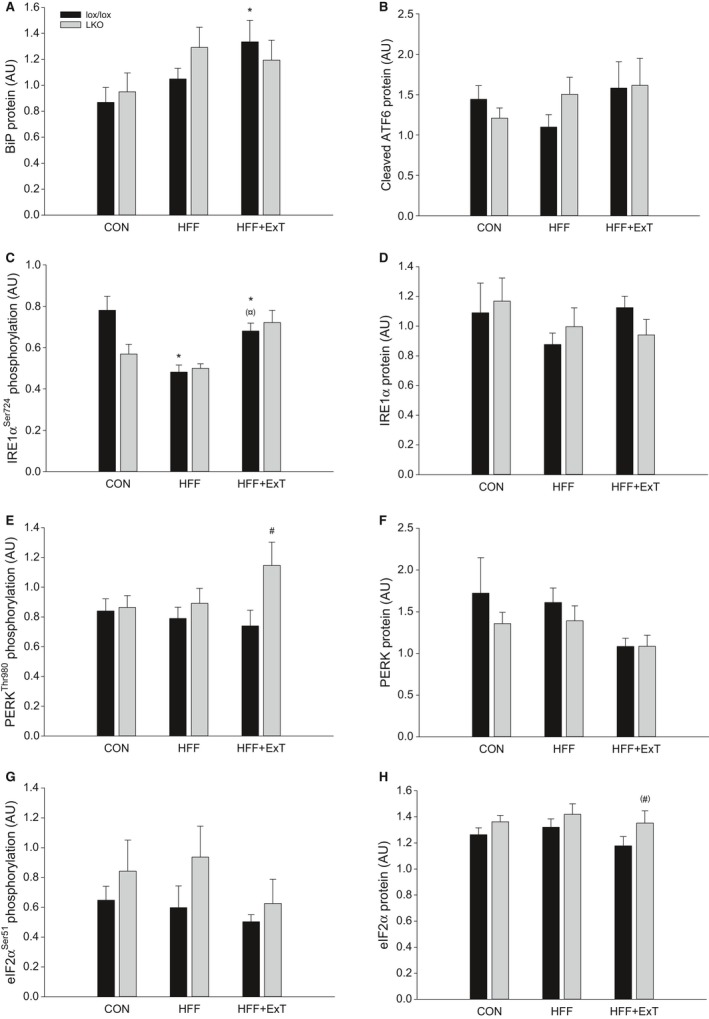
Hepatic BiP protein (A), cleaved ATF6 protein (B), IRE1*α* ^Ser724^ phosphorylation (C), IRE1*α* protein (D), PERK^T^ ^hr980^ phosphorylation (E), PERK protein (F), eIF2*α* ^Ser51^ phosphorylation (G) and eIF2*α* protein (H) content from liver‐specific PGC‐1*α* knockout (LKO) and littermate control (lox/lox) mice fed either a control diet (CON) or a high‐fat high‐fructose diet (HFF) for 13 weeks. Half of the HFF mice performed treadmill exercise training the last 4 weeks (HFF+ExT). Protein content is given in arbitrary units (AU). Values are presented as means ± SE,* n* = 7–10. *Significantly different from CON within given genotype, *P* < 0.05. ^¤^Significantly different from HFF within given genotype, *P* < 0.05. ^#^Significantly different from lox/lox within given group, *P* < 0.05. Parenthesis indicates a tendency for a difference, 0.05 ≤ *P* < 0.1.

BiP protein content in the liver was 1.5 fold higher (*P* < 0.05) in HFF+ExT than CON in lox/lox mice, but otherwise there was no difference between groups. There was no difference between genotypes in any of the groups (Figs. 4A and 6).

There was no difference in cleaved ATF6 protein content in the liver between any of the groups and there was no genotype difference (Figs. 4B and 6).

There was a tendency (*P* = 0.057) for an interaction between groups and genotypes in hepatic IRE1*α* phosphorylation. Hepatic IRE1*α* phosphorylation was in lox/lox mice 40% and 20% lower (*P* < 0.05) in HFF and HFF+ExT, respectively, than in CON mice and tended to be higher (*P* = 0.084) in HFF+ExT than HFF, while there was no difference within LKO mice. There was no difference in phosphorylation of IRE1*α* in the liver between genotypes in any of the groups (Figs. 4C and 6). IRE1*α* protein content was similar between all groups and genotypes (Figs. 4D and 6).

There was no difference in hepatic PERK phosphorylation between groups in either of the genotypes, but the phosphorylation was 1.6 fold higher (*P* < 0.05) in LKO than lox/lox in HFF+ExT (Figs. 4E and 6). There was a tendency (*P* = 0.099) for a difference in hepatic PERK protein content between groups. In addition, there was no difference in hepatic PERK protein content between genotypes in any of the groups (Figs. 4F and 6).

Hepatic eIF2*α* phosphorylation was not different between groups in any of the genotypes. There was a tendency (*P* = 0.098) for a main difference between genotypes (Figs. 4G and 6). There was no difference in eIF2*α* protein content in the liver between any of the groups. In HFF+ExT, hepatic eIF2*α* protein tended to be 1.2 fold higher (*P* = 0.092) in LKO than lox/lox mice (Figs. 4H and 6).

### Downstream UPR mRNA

**Figure 5:**
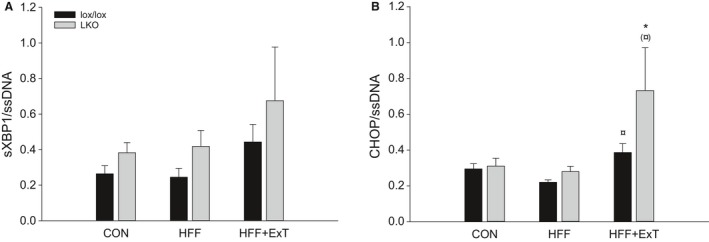
Hepatic spliced (s)XBP1 (A) and CHOP mRNA (B) content in liver from liver‐specific PGC‐1*α* knockout (LKO) and littermate control (lox/lox) mice fed either a control diet (CON) or a high‐fat high‐fructose diet (HFF) for 13 weeks. Half of the HFF fed mice performed treadmill exercise training the last 4 weeks (HFF+ExT). mRNA is normalized to single‐stranded (ss) DNA. Values are presented as means ± SE,* n* = 9–10. *Significantly different from CON within given genotype, *P* < 0.05. ^¤^Significantly different from HFF within given genotype, *P* < 0.05. Parenthesis indicates a tendency for a difference, 0.05 ≤ *P* < 0.1.

There was no effect of HFF or exercise training on hepatic spliced XBP1 mRNA content in either genotype and there was no difference between genotypes in any of the groups (Fig. 5A).

There was no difference in hepatic CHOP mRNA between HFF and CON in either genotype, but CHOP mRNA was in lox/lox mice 1.3 fold higher (*P* < 0.05) in HFF+ExT than HFF and in LKO mice 2.5 fold higher (*P* < 0.05) in HFF+ExT than CON and tended to be higher (*P* = 0.062) in HFF+ExT than HFF. There was no difference in hepatic CHOP mRNA between the genotypes in any of the groups (Fig. 5B).

### HSP72 mRNA, TNF*α* mRNA and JNK phosphorylation

Hepatic HSP72 mRNA content was not different between any of the groups and not different between genotypes (Table 4).

**Table 4:** Hepatic HSP72 mRNA, TNF*α* mRNA and JNK^Thr183/Tyr185^ phosphorylation (phos) normalized to JNK protein content from liver‐specific PGC‐1*α* knockout (LKO) and littermate control (lox/lox) mice fed a control diet (CON) or a high‐fat high‐fructose diet (HFF) for 13 weeks. Half of the HFF mice performed treadmill exercise training the last 4 weeks (HFF+ExT). mRNA is normalized to single‐stranded (ss) DNA. Values are presented as means ± SE, *n* = 8–10

	CON		HFF		HFF+ExT	
	lox/lox	LKO	lox/lox	LKO	lox/lox	LKO
HSP72 mRNA/ssDNA	0.32 ± 0.04	0.30 ± 0.02	0.34 ± 0.04	0.39 ± 0.04	0.34 ± 0.04	0.33 ± 0.05
TNF*α* mRNA/ssDNA	0.18 ± 0.02	0.23 ± 0.03	0.32 ± 0.09	0.28 ± 0.03	0.20 ± 0.02	0.34 ± 0.10
JNK^Thr183/Tyr185^ phos/JNK protein	2.28 ± 0.40	2.47 ± 0.34	2.33 ± 0.35	2.36 ± 0.40	2.34 ± 0.26	2.42 ± 0.26

There was no difference in TNF*α* mRNA between any of the groups and there was no difference between the genotypes (Table 4).

**Figure 6:**
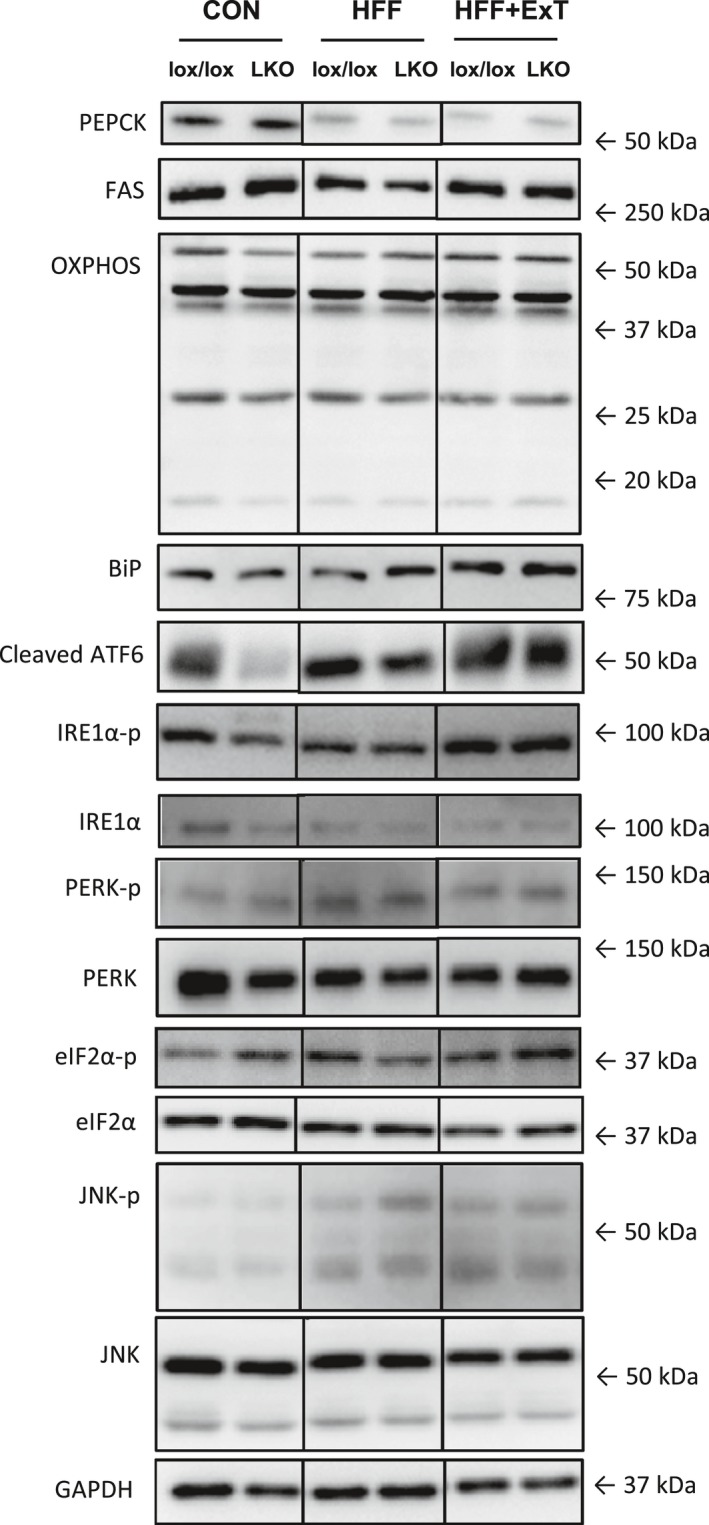
Representative western blots. Phosphoenolpyruvate carboxykinase (PEPCK) protein, fatty acid synthase (FAS) protein, OXPHOS protein, binding immunoglobulin protein (BiP) protein, cleaved activating factor 6 (ATF6) protein, inositol requiring enzyme‐1*α* (IRE1*α*) phosphorylation, IRE1*α* protein, protein kinase RNA‐like ER kinase (PERK) phosphorylation, PERK protein, eukaryotic translation initiation factor 2*α* (eIF2*α*) phosphorylation, eIF2*α* protein, c‐Jun N‐terminal kinase (JNK) phosphorylation, JNK protein and GAPDH protein content in liver from liver‐specific PGC‐1*α* knockout (LKO) and littermate control (lox/lox) mice fed either a control diet (CON) or a high‐fat high‐fructose diet (HFF) for 13 weeks. Half of the HFF fed mice performed treadmill exercise training the last 4 weeks (HFF+ExT). One sample from each experimental group (CON, HFF and HFF+ExT of both genotypes) is represented on each gel. Because samples from mice run acutely were run together with the samples used in the present manuscript, the relevant bands have been cut out of the gel pictures for the representative blots. However, the representative blots show samples run on the same gel.

JNK phosphorylation normalized to JNK protein content was not different between groups and there was no difference between the genotypes (Table 4 and Fig. 6).

## Discussion

The main findings of this study are that diet‐induced triglyceride accumulation in the liver and whole‐body glucose intolerance were accompanied by only modest changes in UPR mainly affecting the IRE1*α* pathway. In addition, exercise training had only minor effects on hepatic UPR and the potential role of PGC‐1*α* in exercise training‐induced regulation of hepatic UPR can therefore not be assessed.

The present observations that body weight, fat percentage, liver weight, and hepatic triglyceride content increased with HFF show that the HFF diet had the expected metabolic impact with accentuated weight gain and hepatic steatosis. In addition, the finding that the plasma glucose AUC in the GTT was higher with HFF than CON indicates that the glucose tolerance was impaired by the HFF. These observations confirm previous findings that rodents fed a HFD with or without fructose supplementation become obese and develop hepatic steatosis and whole‐body glucose intolerance (Gauthier et al. 2003; Koonen et al. 2007; Ren et al. 2012; Schultz et al. 2012). On the other hand, the current observation that the glucose intolerance and hepatic steatosis were not accompanied with any changes in FAS and OXPHOS protein in the liver is not in accordance with previous studies (Koonen et al. 2007; Nadal‐Casellas et al. 2010; Schultz et al. 2012) and indicates that the diet did not challenge the liver enough to regulate hepatic markers of lipogenic and oxidative capacity.

The observation that hepatic PEPCK protein decreased with HFF is in contrast to a previous study reporting increased PEPCK protein content in Swiss mice on 8 weeks of HFD (Souza Pauli et al. 2014), but in accordance with another study in mice on 16 weeks of HFD (Knudsen et al. 2016). Differences in mouse strains or diet composition such as fructose in this study may explain these different observations. The decreased PEPCK protein content in this study indicates that the gluconeogenic capacity was reduced in HFF mice. On the other hand, the finding that HFF mice had increased blood glucose AUC in the PTT compared with CON, may suggest that the gluconeogenic flux in response to the pyruvate injection was elevated in HFF mice. However, it is likely that the removal of the newly produced glucose was lower in HFF than CON, leading to the higher AUC. This possibility is supported by the observation that AUC from the GTT was higher in HFF than in CON.

HFD feeding and triglyceride accumulation in the liver have been associated with ER stress and activation of the UPR (Ozcan et al. 2004; Da Luz et al. 2011; Deldicque et al. 2013; Yuzefovych et al. 2013; Li et al. 2014). The present observation that HFF decreased phosphorylation of IRE1*α* indicates decreased UPR activity and is therefore opposite to the previous findings. In addition, the lack of changes in the PERK and ATF6 pathways of UPR, evidenced by unaltered PERK and eIF2*α* phosphorylation as well as cleaved ATF6 protein content, suggests no induction of hepatic UPR. In line with these observations are the findings that neither of the downstream UPR mRNAs changed with HFF. This is in contrast to previous studies reporting activation of the PERK pathway with increased phosphorylation of PERK and eIF2*α* in mouse and rat liver after 8–16 weeks of HFD feeding (Ozcan et al. 2004; Da Luz et al. 2011; Yuzefovych et al. 2013; Li et al. 2014). IRE1*α* and ATF6 pathways of the UPR were not reported in the previous studies, but activation of the PERK pathway was associated with accumulation of hepatic triglycerides as well as decreased whole‐body glucose tolerance and reduced insulin signaling through Akt in the liver. Other studies have demonstrated increased activation of both the PERK and IRE1*α* pathway in mice fed a HFD for 6 weeks and all three pathways after 16 weeks of HFD (Deldicque et al. 2013; Jiang et al. 2014). These studies also associated the increase in hepatic ER stress with triglyceride accumulation in the liver and decreased whole‐body glucose tolerance. It may be speculated that the diet compositions explain the different observations between the present and previous studies (Deldicque et al. 2013; Jiang et al. 2014), because the diet in the present study also contained fructose. However, other studies feeding mice diets with 35% fructose have shown increased IRE1*α* and/or eIF2*α* phosphorylation paralleled by hepatic triglyceride accumulation and whole‐body glucose intolerance (Montgomery et al. 2015; Wang et al. 2015). Furthermore, the duration of the intervention may explain the discrepancies between the present and previous studies, because the mice in the present study were fed the HFF diet for 13 weeks in contrast to 8 weeks in the previous studies (Montgomery et al. 2015; Wang et al. 2015). Taken together, this study indicates that diet‐induced triglyceride accumulation was not associated with activation of hepatic UPR.

The present finding that triglyceride accumulation in the liver was not associated with elevation of JNK phosphorylation or TNF*α* mRNA content indicates that there was no change in hepatic inflammatory state, which is in contrast to previous studies reporting increased hepatic JNK phosphorylation with HFD in rodents (Samuel et al. 2004; Da Luz et al. 2011; Yuzefovych et al. 2013). This suggests that the HFF‐induced glucose intolerance observed in the present study is not caused by inflammation in the liver as it has previously been reported in obese diabetic mice and HFD fed mice (Nakatani et al. 2004; Samuel et al. 2004). Moreover, it is possible that the observed whole‐body glucose intolerance observed in the current study was caused by reduced glucose removal by skeletal muscle rather than hepatic insulin insensitivity. In addition, further studies are needed to elucidate whether differences between mouse strains or duration of the interventions can explain the different findings.

The present observations that exercise training reversed weight gain and partially reversed the increase in liver weight, fat percentage as well as the impaired glucose tolerance demonstrate that the exercise protocol was effective in eliciting effects on these measurements. However, the lack of change in hepatic triglyceride accumulation with exercise training in lox/lox mice shows that not all parameters were affected. Hence, 4 weeks of exercise training following 9 weeks of HFF feeding appears to have been too short a period for reversing triglyceride accumulation in the liver. Furthermore, because the diet‐induced glucose intolerance was partially reversed by exercise training it seems that the impaired glucose tolerance in this study was not caused by hepatic triglyceride accumulation.

The present finding that exercise training had only minor effects on the investigated UPR markers with elevated BiP protein and CHOP mRNA content relative to CON and HFF mice, respectively, and a tendency for an increase in IRE1*α* phosphorylation compared with HFF is in contrast to previous studies (Chapados and Lavoie 2010; Da Luz et al. 2011). In rats, it was demonstrated that HFD‐induced phosphorylation of PERK, eIF2*α* and JNK as well as impairment of Akt signaling in the liver was prevented by concomitant swimming exercise training (Da Luz et al. 2011). Another study in rats reported increased hepatic BiP protein in response to treadmill exercise training performed simultaneously with HFD feeding compared with HFD fed sedentary mice (Chapados and Lavoie 2010), and this is also in contrast to the current study. In addition, it should be noted that the present study cannot assess the potential ability of exercise training to reverse HFF‐induced changes in hepatic UPR due to the only limited changes in UPR with the HFF intervention.

The current observation that the protein content of PEPCK and OXPHOS was unchanged with exercise training is in contrast to a previous study reporting increased PEPCK, Cyt c, and COXIV protein content in mouse liver after 5 weeks of combined treadmill running and voluntary wheel running exercise training (Haase et al. 2011). The HFF diet in the present study may have caused this discrepancy by masking exercise training effects. This possibility is supported by the previous observation that 16 weeks of wheel running exercise training did not either affect hepatic PEPCK and OXPHOS protein content in mice on HFD (Knudsen et al. 2016).

The present finding that lack of PGC‐1*α* in the liver did not affect hepatic UPR in CON mice is in contrast to a previous study reporting increased BiP protein content in the liver of whole‐body PGC‐1*α* knockout mice compared with wildtypes (Kristensen et al. 2017) and suggests that PGC‐1*α* is not required for maintaining basal UPR. On the other hand, the observation that PERK phosphorylation increased in HFF+ExT within LKO, but not lox/lox mice indicates that hepatic PGC‐1*α* is involved in regulation of the PERK pathway in response to exercise training. In addition, the elevated hepatic PERK phosphorylation in LKO mice with exercise training suggests that the LKO mice were more challenged by the exercise training than lox/lox mice leading to activation of PERK. However, the potential requirement of PGC‐1*α* in regulating hepatic UPR in response to exercise training is not possible to assess in this study, because exercise training only induced minor changes in hepatic UPR.

The present findings that hepatic PEPCK protein and plasma *β*‐hydroxubutyrate as well as plasma and liver triglyceride content were similar in LKO and lox/lox mice were not as hypothesized and not in accordance with previous observations. Thus, previous studies have reported increased PEPCK protein in the liver of whole‐body PGC‐1*α* knockout mice (Lin et al. 2004; Haase et al. 2011; Kristensen et al. 2017). Furthermore, plasma and liver triglyceride content has previously been shown to be lower in liver of adenoviral PGC‐1*α* overexpression rats than controls (Morris et al. 2012) and plasma triglycerides and *β*‐hydroxybutyrate to be increased and decreased, respectively, in heterozygous liver‐specific PGC‐1*α* knockout mice (Estall et al. 2009). However, these differences between the present and previous studies may be explained by the use of different animal models because this study used homozygous liver‐specific PGC‐1*α* knockout mice, whereas mice in the previous studies were whole‐body PGC‐1*α* knockout mice (Lin et al. 2004; Haase et al. 2011; Kristensen et al. 2017) and heterozygous liver‐specific PGC‐1*α* knockout mice (Estall et al. 2009), while the study by Morris et al. (2012) was performed in rats.

The observation that glucose and pyruvate tolerance was similar in the two genotypes is in accordance with observations in heterozygous liver PGC‐1*α* knockout mice (Estall et al. 2009) and indicates that regulation of glucose homeostasis was independent of PGC‐1*α* in the liver. Another study, however, reported increased glucose tolerance in PGC‐1*α* whole‐body knockout mice compared to wild‐type mice when fed both chow and HFD (Lin et al. 2004). Similarly, heterozygous liver‐specific PGC‐1*α* mice on HFD have been shown to have decreased glucose AUC following a PTT relative to control mice (Estall et al. 2009). Different knockout models may explain the discrepancies, which highlights the challenge that different animal models may indicate different roles of PGC‐1*α*.

In conclusion, this study provides evidence for dissociation between diet‐induced hepatic triglyceride accumulation and UPR activation in the liver. In addition, hepatic PGC‐1*α* was not required for maintaining basal UPR. However, it is not possible to fully elucidate the ability of exercise training to reverse HFF‐induced changes in UPR as well as the requirement of PGC‐1*α* for exercise training‐induced adaptations in hepatic UPR, because only minor changes were observed in hepatic UPR with HFF and exercise training.

## Conflict of Interest

The authors have no conflicts of interest.
